# Supplementary material for: Integrative modeling of tumor genomes and epigenomes for enhanced cancer diagnosis by cell-free DNA
Source: Nat Commun. 2023 Apr 10;14:2017. doi: 10.1038/s41467-023-37768-3 (PMC10085982; doi:10.1038/s41467-023-37768-3)
Supplement: Supplementary file 1 — Supplementary Information [file 41467_2023_37768_MOESM1_ESM.pdf]

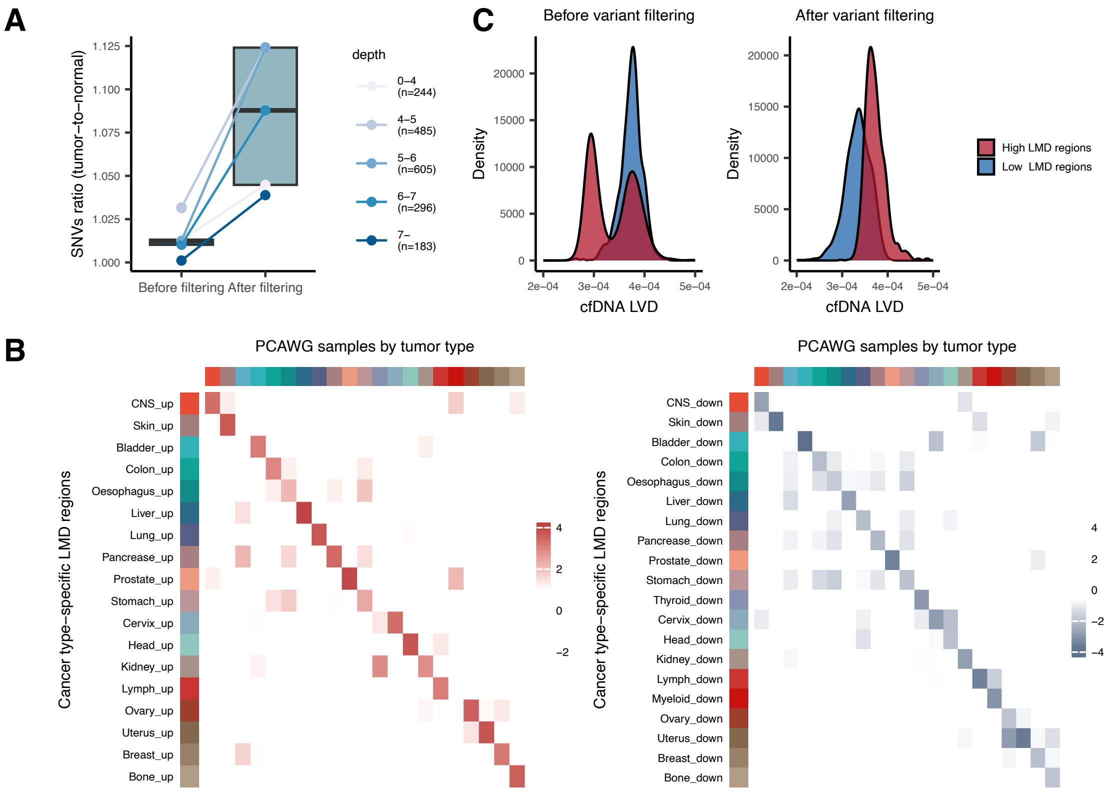
A
SNVs ratio (tumor−to−normal)
1.125
1.100
1.075
1.050
1.025
1.000
Before filtering After filtering
depth
0–4 (n=244)
4–5 (n=485)
5–6 (n=605)
6–7 (n=296)
7– (n=183)
C
Before variant filtering
After variant filtering
Density
20000
15000
10000
5000
0
2e−04
3e−04
4e−04
5e−04
cfDNA LVD
High LMD regions
Low LMD regions
B
PCAWG samples by tumor type
Cancer type−specific LMD regions
CNS_up
Skin_up
Bladder_up
Colon_up
Oesophagus_up
Liver_up
Lung_up
Pancrease_up
Prostate_up
Stomach_up
Cervix_up
Head_up
Kidney_up
Lymph_up
Ovary_up
Uterus_up
Breast_up
Bone_up
4
2
0
−2
PCAWG samples by tumor type
Cancer type−specific LMD regions
CNS_down
Skin_down
Bladder_down
Colon_down
Oesophagus_down
Liver_down
Lung_down
Pancrease_down
Prostate_down
Stomach_down
Thyroid_down
Cervix_down
Head_down
Kidney_down
Lymph_down
Myeloid_down
Ovary_down
Uterus_down
Breast_down
Bone_down
4
2
0
−2
−4

Supplementary Fig. 1. Supporting data for genome model development. (A) The ratio of single nucleotide variants detected in cancer versus normal cfDNA samples before and after a pplying our variant filtering pipeline. Variant calling was performed at differing read depths. E ach box indicates IQR and median, whiskers indicates 1.5 x IQR, black dots indicates outlier. **(B)** Heatmaps showing the average LMD values in high LMD regions (left) and low LMD regio ns (right), specifically identified for each cancer type. The color intensity is scaled to show th e degree of relative mutation enrichment (left) or mutation depletion (left). A total of 2,754 PCAWG samples were used for this analysis. **(C)** Distribution of the LVD in cfDNA in the abo ve identified high or low LMD regions in the matching cancer type before and after applying our variant filtering pipeline. Source data are provided as a Source Data file.

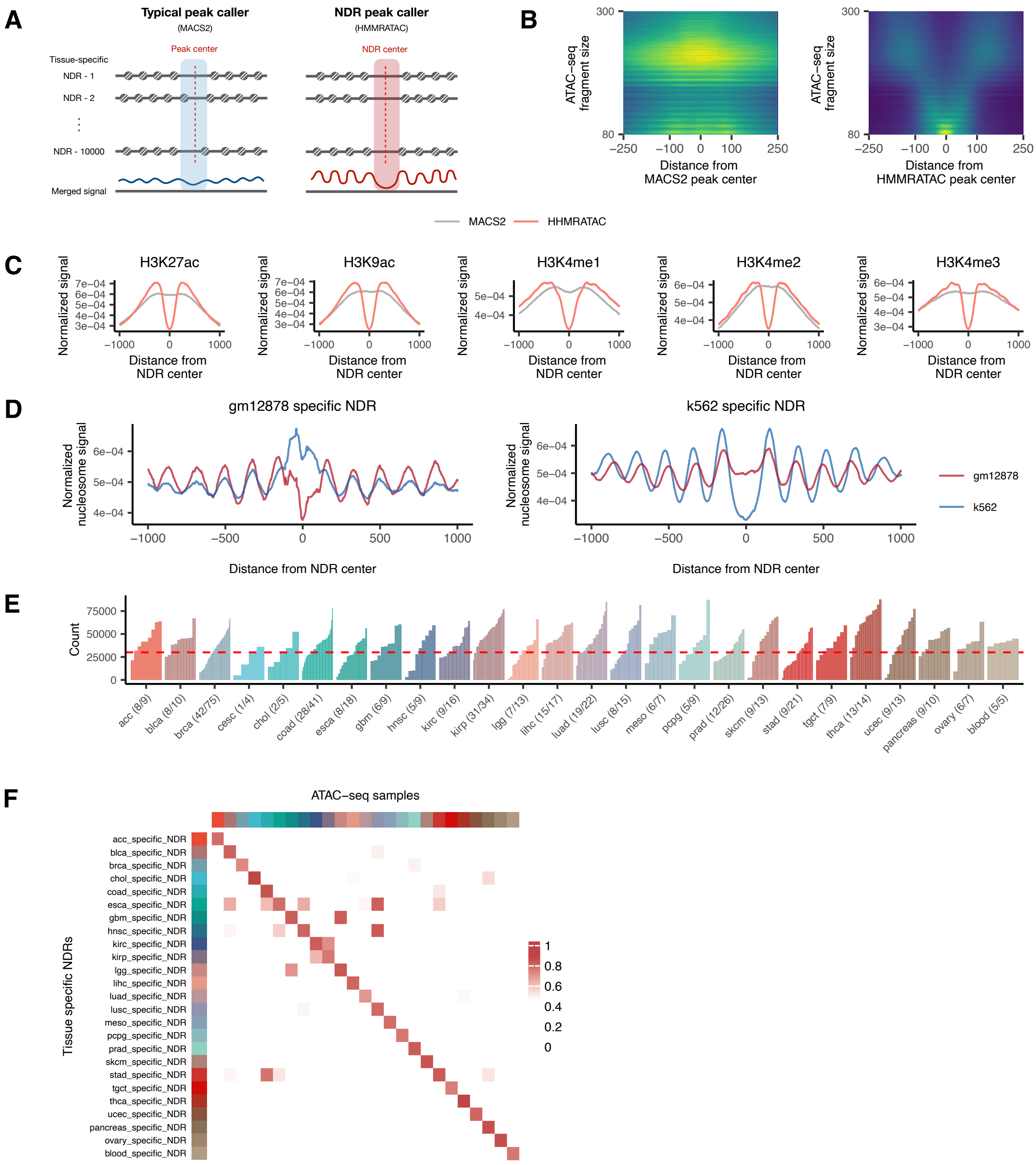
A
Typical peak caller
(MACS2)
NDR peak caller
(HMMRATAC)
Peak center
NDR center
Tissue-specific
NDR - 1
NDR - 2
NDR - 10000
Merged signal
B
ATAC−seq fragment size
Distance from MACS2 peak center
Distance from HMMRATAC peak center
MACS2
HHMRATAC
C
H3K27ac
H3K9ac
H3K4me1
H3K4me2
H3K4me3
Normalized signal
Distance from NDR center
D
gm12878 specific NDR
k562 specific NDR
Normalized nucleosome signal
Distance from NDR center
gm12878
k562
E
Count
acc (8/9)
blca (8/10)
brca (42/75)
cesc (1/4)
chol (2/5)
coad (28/41)
esca (8/18)
gbm (6/9)
hnsc (5/9)
kirc (9/16)
kirp (31/34)
lgg (7/13)
lihc (15/17)
luad (19/22)
lusc (8/15)
meso (6/7)
pcpg (5/9)
prad (12/26)
skcm (9/13)
stad (9/21)
tgct (7/9)
thca (13/14)
ucec (9/13)
pancreas (9/10)
ovary (6/7)
blood (5/5)
F
ATAC−seq samples
Tissue specific NDRs
acc_specific_NDR
blca_specific_NDR
brca_specific_NDR
chol_specific_NDR
coad_specific_NDR
esca_specific_NDR
gbm_specific_NDR
hnsc_specific_NDR
kirc_specific_NDR
kirp_specific_NDR
lgg_specific_NDR
lihc_specific_NDR
luad_specific_NDR
lusc_specific_NDR
meso_specific_NDR
pcpg_specific_NDR
prad_specific_NDR
skcm_specific_NDR
stad_specific_NDR
tgct_specific_NDR
thca_specific_NDR
ucec_specific_NDR
pancreas_specific_NDR
ovary_specific_NDR
blood_specific_NDR

Supplementary Fig. 2. Supporting data for epigenome model development. (A) Illustration of peak call process for typical peak caller (MACS2) and NDR peak caller (HMMRATAC). **(B)** V-plot image constructed for the NDRs identified by ATAC-seq peak calling by MACS2 (left) and HMMRATAC (right). Data from the GM12878 cell line are shown. **(C)** ChIP-seq signals of histone modification (H3K27ac, H3K9ac, H3K4me1, H3K4me2, and H3K4me3) centered on the NDRs identified by HMMRATAC (red) and MACS2 (gray). **(D)** Nucleosome occupancy at GM12878-specific (left) and K562-specific (right) NDRs in GM12878 cell line (red) and in K562 cell line (blue). **(E)** The number of the NDRs identified by HMMRATAC in 431 samples grouped into 23 tumor tissues, 2 normal tissues, or PBMCs. The red horizontal dashed line indicates the peak count threshold for sample filtering. The filtered and total number of samples is denoted in parentheses. **(F)** Heatmap showing the average peak scores of the NDRs specific to each tissue group. The color intensity is proportional to the peak score. Source data are provided as a Source Data file.

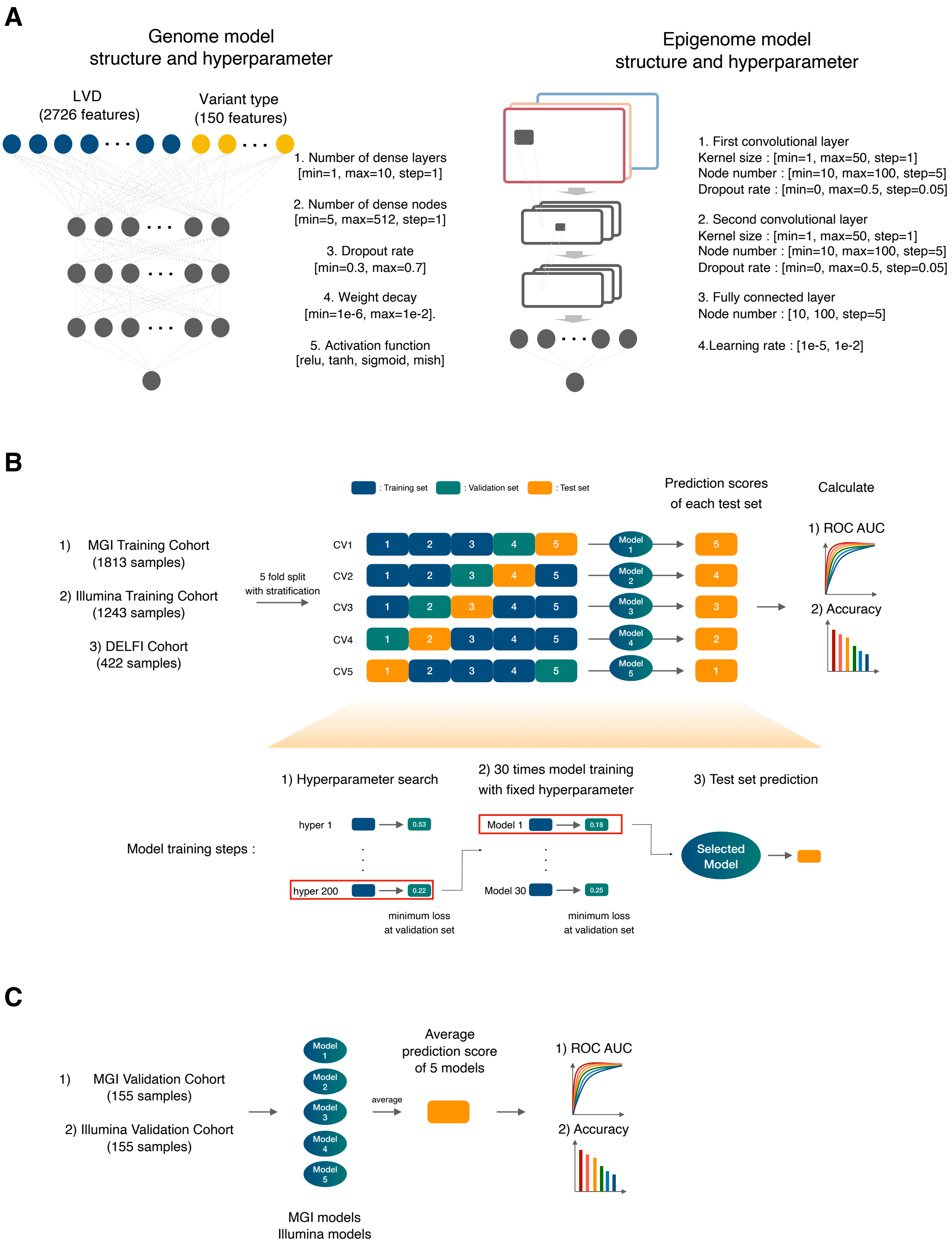

A
Genome model
structure and hyperparameter
LVD
(2726 features)
Variant type
(150 features)
1. Number of dense layers
[min=1, max=10, step=1]
2. Number of dense nodes
[min=5, max=512, step=1]
3. Dropout rate
[min=0.3, max=0.7]
4. Weight decay
[min=1e-6, max=1e-2].
5. Activation function
[relu, tanh, sigmoid, mish]
Epigenome model
structure and hyperparameter
1. First convolutional layer
Kernel size : [min=1, max=50, step=1]
Node number : [min=10, max=100, step=5]
Dropout rate : [min=0, max=0.5, step=0.05]
2. Second convolutional layer
Kernel size : [min=1, max=50, step=1]
Node number : [min=10, max=100, step=5]
Dropout rate : [min=0, max=0.5, step=0.05]
3. Fully connected layer
Node number : [10, 100, step=5]
4.Learning rate : [1e-5, 1e-2]
B
: Training set
: Validation set
: Test set
Prediction scores
of each test set
Calculate
1) MGI Training Cohort
(1813 samples)
2) Illumina Training Cohort
(1243 samples)
3) DELFI Cohort
(422 samples)
5 fold split
with stratification
CV1
CV2
CV3
CV4
CV5
Model 1
Model 2
Model 3
Model 4
Model 5
1) ROC AUC
2) Accuracy
1) Hyperparameter search
2) 30 times model training
with fixed hyperparameter
3) Test set prediction
Model training steps :
hyper 1
0.53
hyper 200
0.22
minimum loss
at validation set
Model 1
0.18
Model 30
0.25
minimum loss
at validation set
Selected
Model
C
1) MGI Validation Cohort
(155 samples)
2) Illumina Validation Cohort
(155 samples)
Model 1
Model 2
Model 3
Model 4
Model 5
MGI models
Illumina models
average
Average
prediction score
of 5 models
1) ROC AUC
2) Accuracy

Supplementary Fig. 3. Schematic of the training and prediction of genome and epigenome models. **(A)** Illustration of the genome and epigenome model structure and hyperparameter space. **(B)** Schematic of the model training process using the training cohorts. Stratified five-fold cross-validation method was used to train and evaluate the models for each training cohort. At each split, the best hyperparameter that minimizes the validation loss was selected. Using the fixed hyperparameter, the model was trained 30 times and the model with lowest validation loss was chosen as the final model. **(C)** Schematic of the external prediction using the validation cohorts. The validation cohorts were predicted using the five models trained with the training cohort. The average of five prediction values was used as the final prediction score.

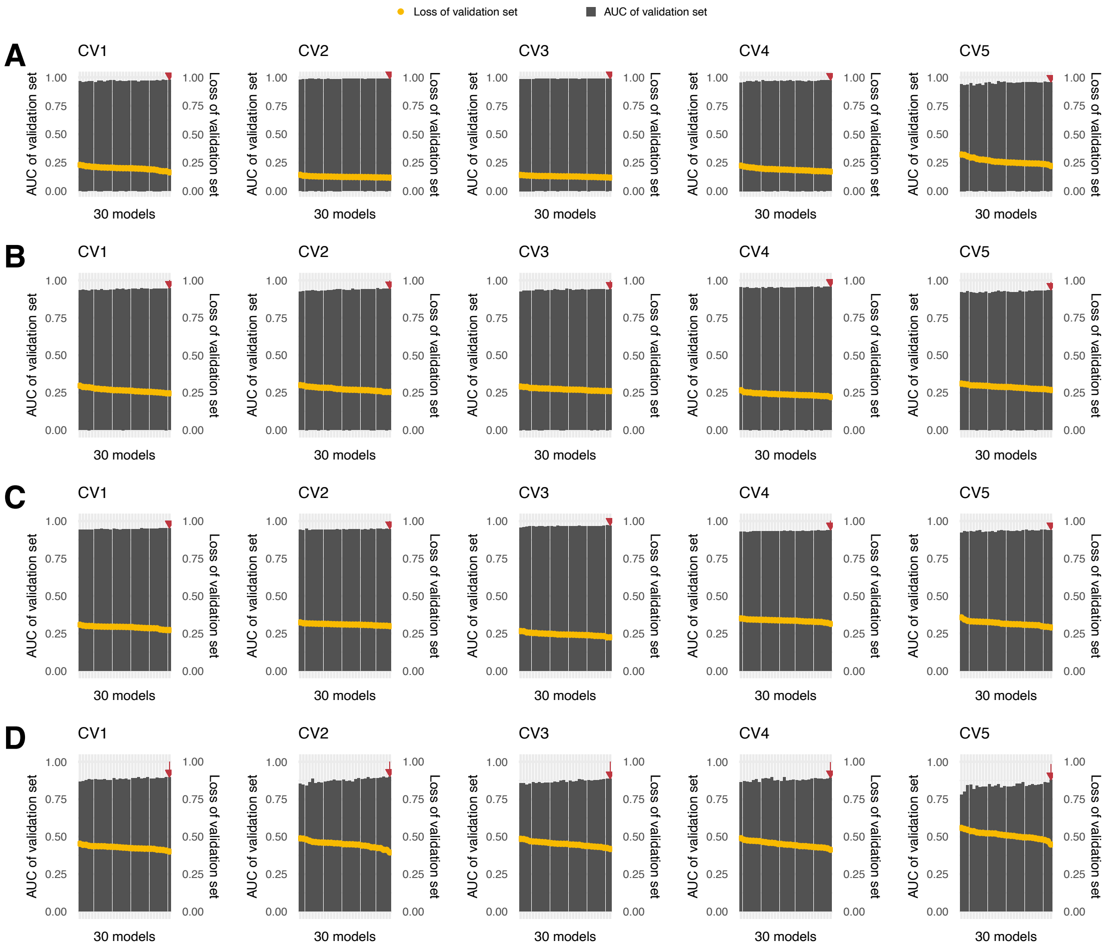

Loss of validation set
AUC of validation set
A
B
C
D
CV1
CV2
CV3
CV4
CV5
AUC of validation set
Loss of validation set
30 models

Supplementary Fig. 4. Validation performance of the cancer detection models trained wit h fixed hyperparameter in 30 different random states. (A-D) Validation loss and ROC-AUC of **(A)** the genome model of the MGI training cohort, **(B)** the epigenome model of the MGI t raining cohort, **(C)** the genome model of the Illumina training cohort, **(D)** the epigenome mo del of the Illumina training cohort. Bar plots (gray) represent ROC AUC of the validation set. Line plot (yellow) represent loss of the validation set. The red arrows indicate the final mode l with the minimum loss at the validation set. AUC, area under the curve; CV, cross validatio n. Source data are provided as a Source Data file.

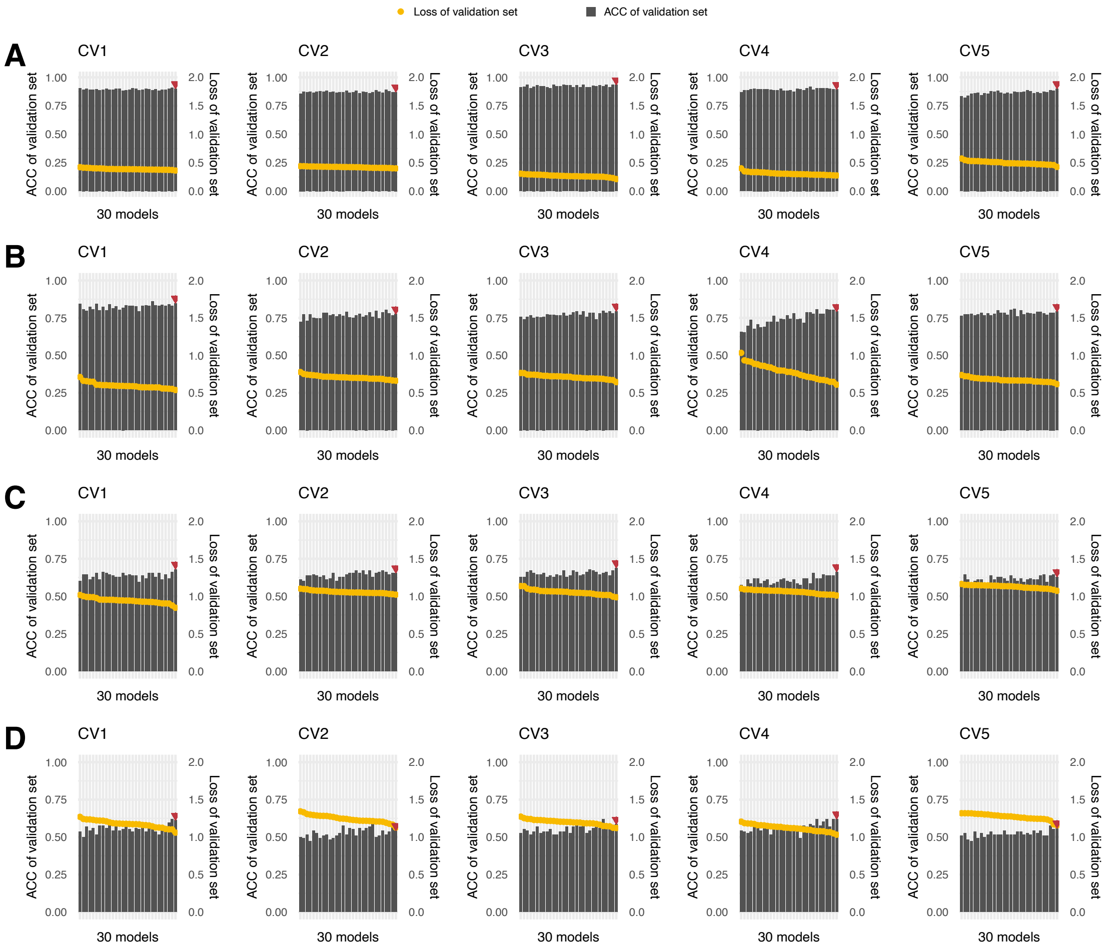
Loss of validation set
ACC of validation set
A
B
C
D
CV1
CV2
CV3
CV4
CV5
ACC of validation set
Loss of validation set
30 models

Supplementary Fig. 5. Validation performance of the cancer localization models trained with fixed hyperparameter in 30 different random states. (A-D) Validation accuracy and lo ss of **(A)** the genome model of the MGI training cohort, **(B)** the epigenome model of the MG I training cohort, **(C)** the genome model of the Illumina training cohort and **(D)** the epigeno me model of the Illumina training cohort. Bar plots (gray) represent accuracy of the validatio n set. Line plot (yellow) represent loss of the validation set. The red arrows indicate the final model with the minimum loss at the validation set. ACC, accuracy; CV, cross-validation. Sour ce data are provided as a Source Data file.

A

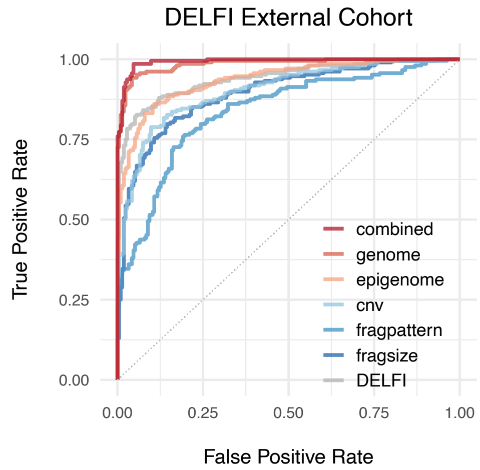

B

DELFI	External Cohort	
	auc	95% CI
combined	0.993	0.987-0.998
genome	0.986	0.976-0.993
epigenome	0.937	0.913-0.957
cnv	0.903	0.875-0.932
fragpattern	0.838	0.798-0.874
fragsize	0.893	0.863-0.923
DELFI	0.941	0.916-0.962

C

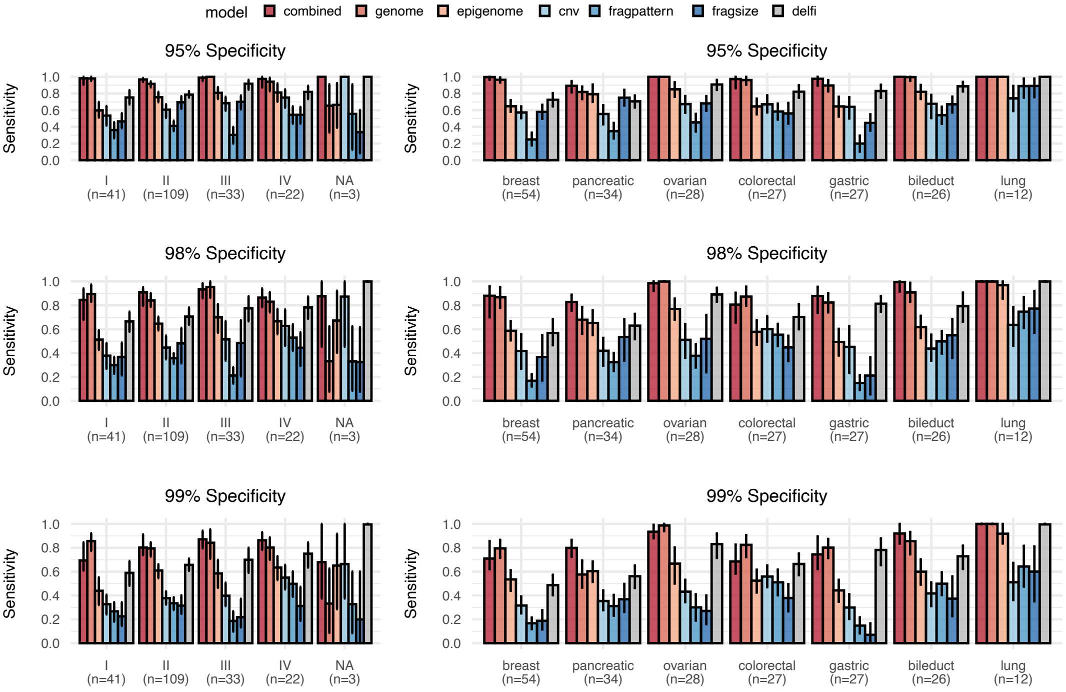

Supplementary Fig. 6. Performance of cancer detection on an external cohort. (A) ROC c urve and **(B)** ROC-AUC table providing the 95% confidence interval for different models on th e DELFI dataset[9]. A total of 208 cacner and 214 normal control samples were used. **(C)** Sensit ivity values with the 95% confidence interval at 95%, 98%, and 99% specificity broken down by the tumor stage (left) and cancer type (right) for the DELFI dataset[9]. Confidence interval f or sensitivity value was calculated from 1,000 bootstraping samplings. **(A-C)** Our genome, epig enome, and combined models were compared with predictions based on fragmentation pattern s[9] (fragpattern), fragment size profiles[8] (fragsize), and copy number variations[3] (cnv). The gray ROC curve and bar graphs marked as DELFI correspond to the score provided by the authors[9] combining fragmentation with other features. auc, area under the curve; NA, stage informati on not available; CI, Confidence interval. Source data are provided as a Source Data file.

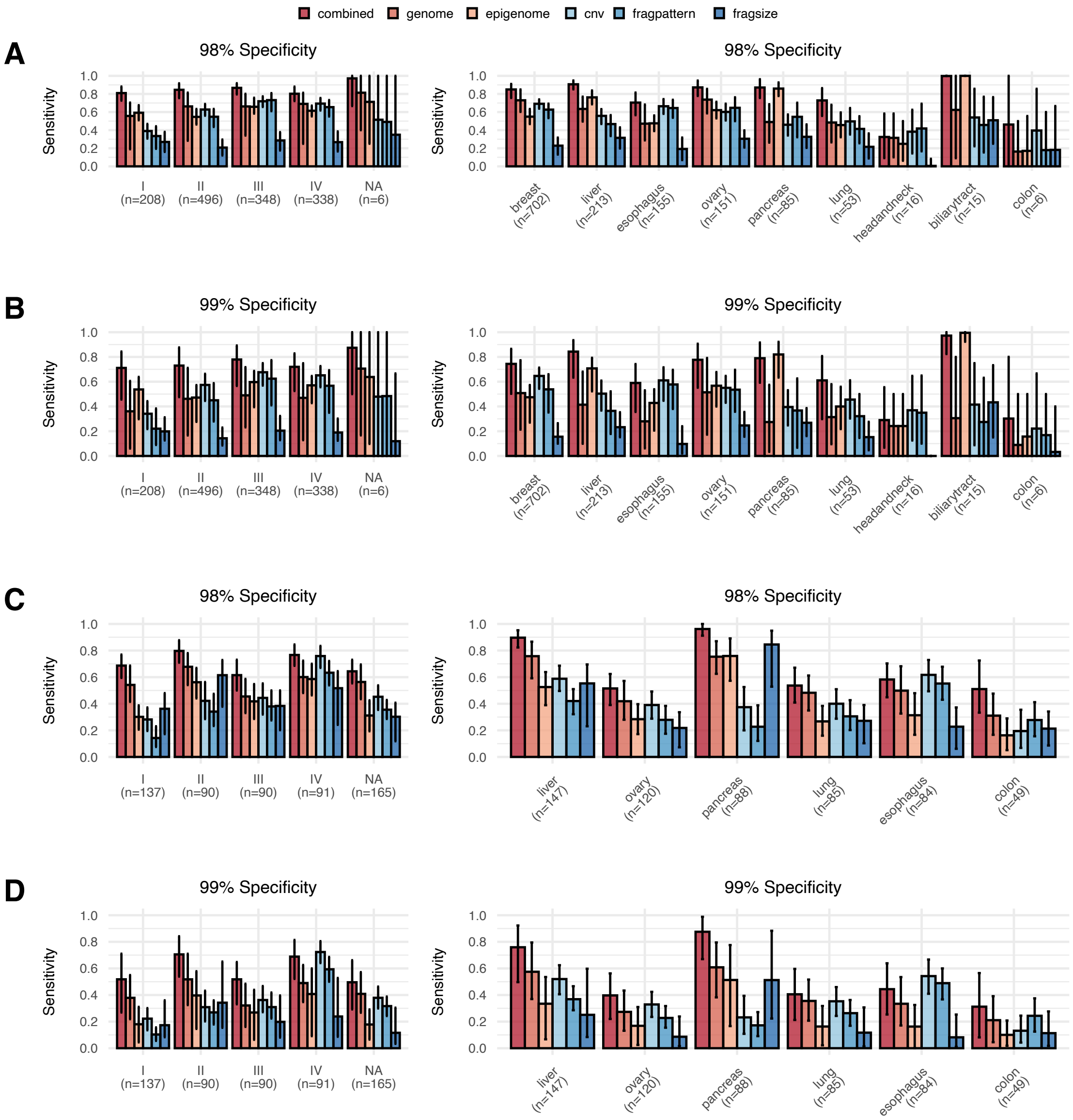

combined
genome
epigenome
cnv
fragpattern
fragsize
A
B
C
D
98% Specificity
99% Specificity
Sensitivity
I (n=208)
II (n=496)
III (n=348)
IV (n=338)
NA (n=6)
breast (n=702)
liver (n=213)
esophagus (n=155)
ovary (n=151)
pancreas (n=85)
lung (n=53)
headandneck (n=16)
biliarytract (n=15)
colon (n=6)
I (n=137)
II (n=90)
III (n=90)
IV (n=91)
NA (n=165)
liver (n=147)
ovary (n=120)
pancreas (n=88)
lung (n=85)
esophagus (n=84)
colon (n=49)

Supplementary Fig. 7. Sensitivity of cancer detection. (A-B) Sensitivity values with the 95% confidence interval at **(A)** 98% and **(B)** 99% specificity broken down by the tumor stage (left) and cancer type (right) on the MGI training cohort. **(C-D)** Sensitivity values with the 95% confidence interval at **(C)** 98% and **(D)** 99% specificity broken down by the tumor stage (left) and cancer type (right) on the Illumina training cohort. Confidence interval for sensitivity value was calculated from 1,000 bootstraping samplings. Our genome, epigenome, and combined models were compared with predictions based on fragmentation patterns[9] (fragpattern), fragment size profiles[8] (fragsize), and copy number variations[3] (cnv). NA, stage information not available. Source data are provided as a Source Data file.

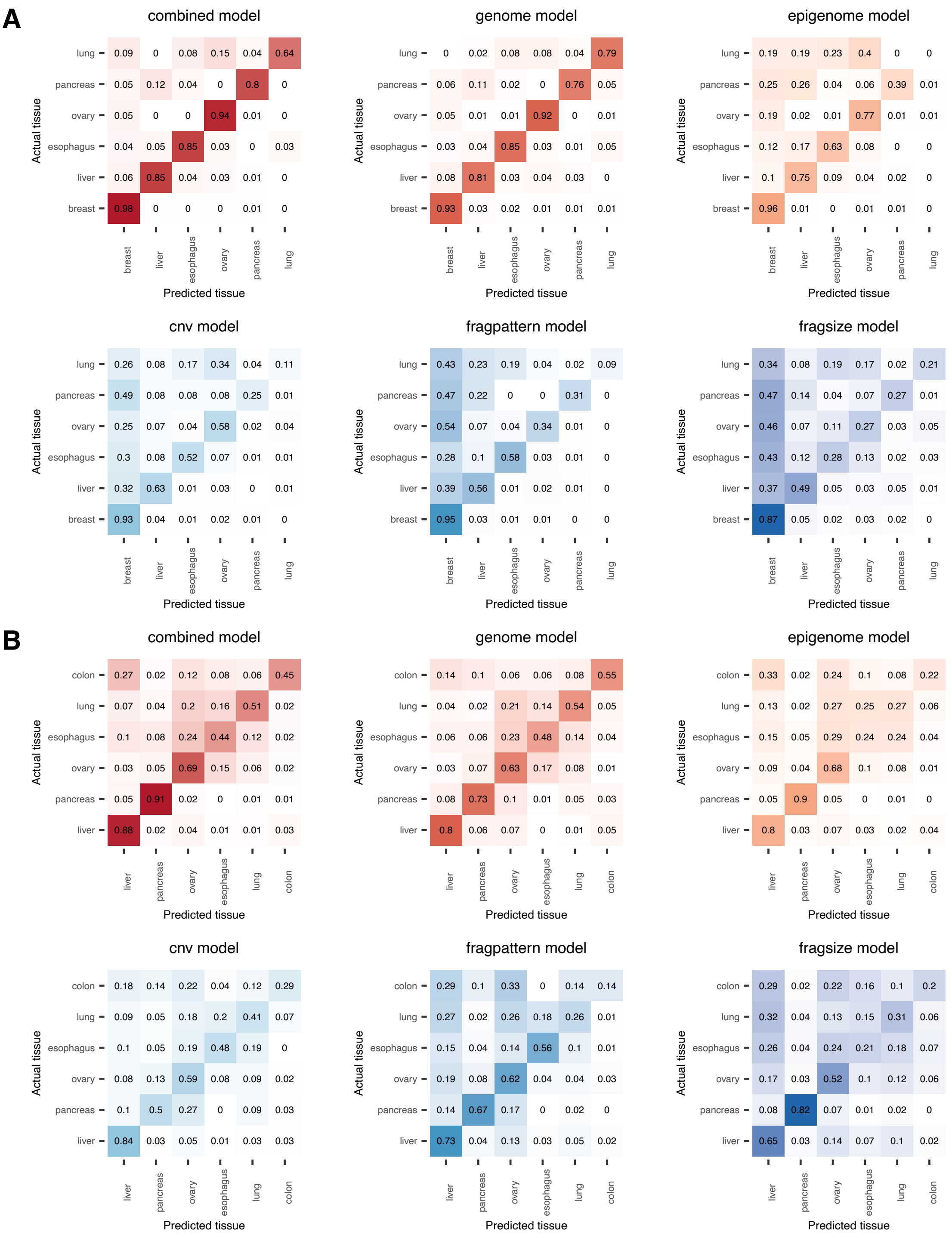
A
combined model
lung 0.09 0 0.08 0.15 0.04 0.64
pancreas 0.05 0.12 0.04 0 0.8 0
ovary 0.05 0 0 0.94 0.01 0
esophagus 0.04 0.05 0.85 0.03 0 0.03
liver 0.06 0.85 0.04 0.03 0.01 0
breast 0.98 0 0 0 0.01 0
breast liver esophagus ovary pancreas lung
Actual tissue
Predicted tissue
genome model
lung 0 0.02 0.08 0.08 0.04 0.79
pancreas 0.06 0.11 0.02 0 0.76 0.05
ovary 0.05 0.01 0.01 0.92 0 0.01
esophagus 0.03 0.04 0.85 0.03 0.01 0.05
liver 0.08 0.81 0.03 0.04 0.03 0
breast 0.93 0.03 0.02 0.01 0.01 0.01
breast liver esophagus ovary pancreas lung
Actual tissue
Predicted tissue
epigenome model
lung 0.19 0.19 0.23 0.4 0 0
pancreas 0.25 0.26 0.04 0.06 0.39 0.01
ovary 0.19 0.02 0.01 0.77 0.01 0.01
esophagus 0.12 0.17 0.63 0.08 0 0
liver 0.1 0.75 0.09 0.04 0.02 0
breast 0.96 0.01 0 0.01 0.01 0
breast liver esophagus ovary pancreas lung
Actual tissue
Predicted tissue
cnv model
lung 0.26 0.08 0.17 0.34 0.04 0.11
pancreas 0.49 0.08 0.08 0.08 0.25 0.01
ovary 0.25 0.07 0.04 0.58 0.02 0.04
esophagus 0.3 0.08 0.52 0.07 0.01 0.01
liver 0.32 0.63 0.01 0.03 0 0.01
breast 0.93 0.04 0.01 0.02 0.01 0
breast liver esophagus ovary pancreas lung
Actual tissue
Predicted tissue
fragpattern model
lung 0.43 0.23 0.19 0.04 0.02 0.09
pancreas 0.47 0.22 0 0 0.31 0
ovary 0.54 0.07 0.04 0.34 0.01 0
esophagus 0.28 0.1 0.58 0.03 0.01 0
liver 0.39 0.56 0.01 0.02 0.01 0
breast 0.95 0.03 0.01 0.01 0 0
breast liver esophagus ovary pancreas lung
Actual tissue
Predicted tissue
fragsize model
lung 0.34 0.08 0.19 0.17 0.02 0.21
pancreas 0.47 0.14 0.04 0.07 0.27 0.01
ovary 0.46 0.07 0.11 0.27 0.03 0.05
esophagus 0.43 0.12 0.28 0.13 0.02 0.03
liver 0.37 0.49 0.05 0.03 0.05 0.01
breast 0.87 0.05 0.02 0.03 0.02 0
breast liver esophagus ovary pancreas lung
Actual tissue
Predicted tissue
B
combined model
colon 0.27 0.02 0.12 0.08 0.06 0.45
lung 0.07 0.04 0.2 0.16 0.51 0.02
esophagus 0.1 0.08 0.24 0.44 0.12 0.02
ovary 0.03 0.05 0.69 0.15 0.06 0.02
pancreas 0.05 0.91 0.02 0 0.01 0.01
liver 0.88 0.02 0.04 0.01 0.01 0.03
liver pancreas ovary esophagus lung colon
Actual tissue
Predicted tissue
genome model
colon 0.14 0.1 0.06 0.06 0.08 0.55
lung 0.04 0.02 0.21 0.14 0.54 0.05
esophagus 0.06 0.06 0.23 0.48 0.14 0.04
ovary 0.03 0.07 0.63 0.17 0.08 0.01
pancreas 0.08 0.73 0.1 0.01 0.05 0.03
liver 0.8 0.06 0.07 0 0.01 0.05
liver pancreas ovary esophagus lung colon
Actual tissue
Predicted tissue
epigenome model
colon 0.33 0.02 0.24 0.1 0.08 0.22
lung 0.13 0.02 0.27 0.25 0.27 0.06
esophagus 0.15 0.05 0.29 0.24 0.24 0.04
ovary 0.09 0.04 0.68 0.1 0.08 0.01
pancreas 0.05 0.9 0.05 0 0.01 0
liver 0.8 0.03 0.07 0.03 0.02 0.04
liver pancreas ovary esophagus lung colon
Actual tissue
Predicted tissue
cnv model
colon 0.18 0.14 0.22 0.04 0.12 0.29
lung 0.09 0.05 0.18 0.2 0.41 0.07
esophagus 0.1 0.05 0.19 0.48 0.19 0
ovary 0.08 0.13 0.59 0.08 0.09 0.02
pancreas 0.1 0.5 0.27 0 0.09 0.03
liver 0.84 0.03 0.05 0.01 0.03 0.03
liver pancreas ovary esophagus lung colon
Actual tissue
Predicted tissue
fragpattern model
colon 0.29 0.1 0.33 0 0.14 0.14
lung 0.27 0.02 0.26 0.18 0.26 0.01
esophagus 0.15 0.04 0.14 0.56 0.1 0.01
ovary 0.19 0.08 0.62 0.04 0.04 0.03
pancreas 0.14 0.67 0.17 0 0.02 0
liver 0.73 0.04 0.13 0.03 0.05 0.02
liver pancreas ovary esophagus lung colon
Actual tissue
Predicted tissue
fragsize model
colon 0.29 0.02 0.22 0.16 0.1 0.2
lung 0.32 0.04 0.13 0.15 0.31 0.06
esophagus 0.26 0.04 0.24 0.21 0.18 0.07
ovary 0.17 0.03 0.52 0.1 0.12 0.06
pancreas 0.08 0.82 0.07 0.01 0.02 0
liver 0.65 0.03 0.14 0.07 0.1 0.02
liver pancreas ovary esophagus lung colon
Actual tissue
Predicted tissue

Supplementary Fig. 8. Confusion matrix for tissue-of-origin localization on the (A) MGI training cohort and (B) Illumina training cohort. The y-axis represents the actual site, and the x-axis represents the predicted site. The numbers in the cells of the matrix represent th e proportion of samples of each cancer type localized to respective tumor sites. Our genome, epigenome, and combined models were compared with predictions based on fragmentation pat terns[9] (fragpattern), fragment size profiles[8] (fragsize), and copy number variations[3] (cnv). Sourc e data are provided as a Source Data file.

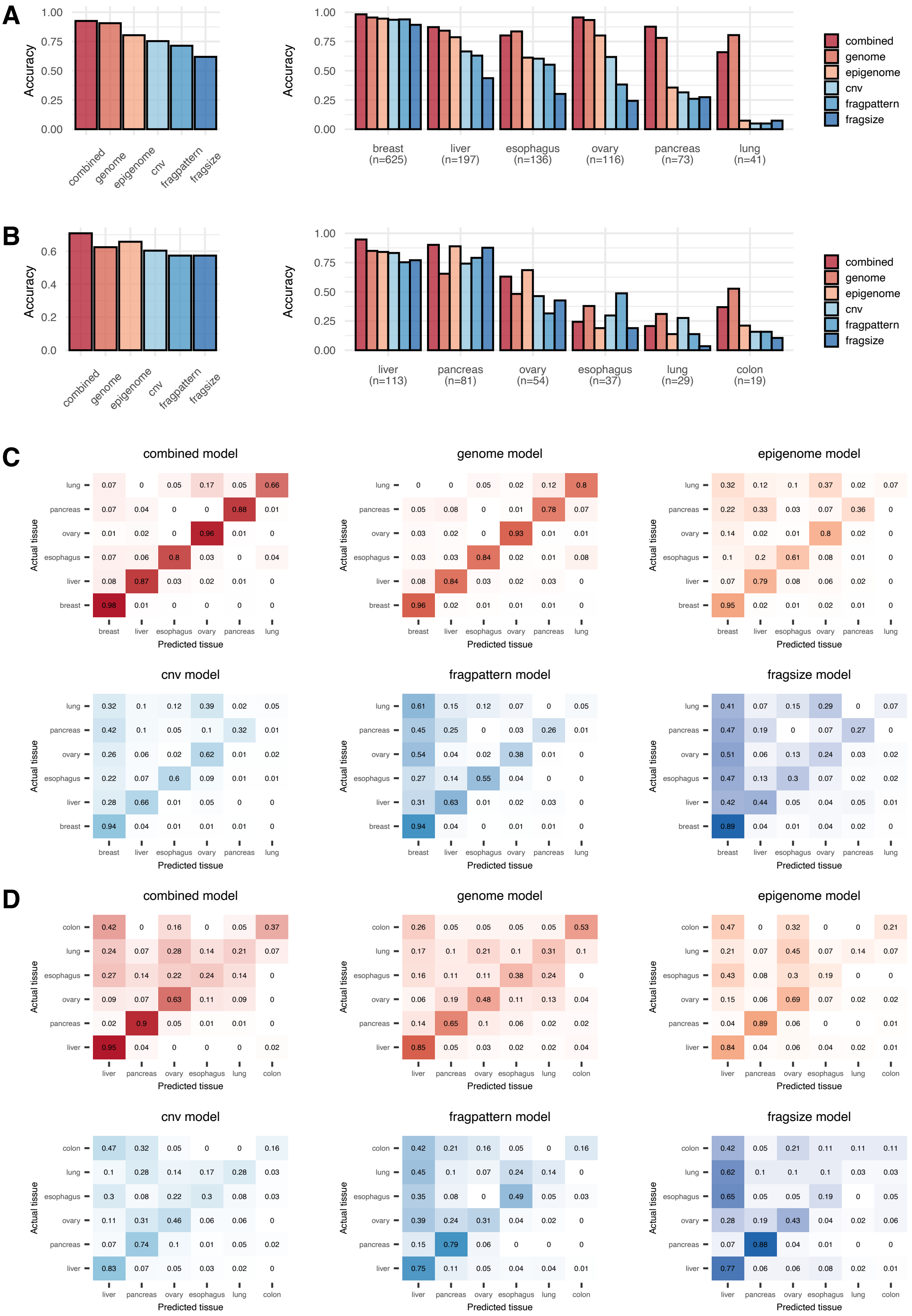
A
B
C
D
Accuracy
combined
genome
epigenome
cnv
fragpattern
fragsize
breast (n=625)
liver (n=197)
esophagus (n=136)
ovary (n=116)
pancreas (n=73)
lung (n=41)
liver (n=113)
pancreas (n=81)
ovary (n=54)
esophagus (n=37)
lung (n=29)
colon (n=19)
combined model
genome model
epigenome model
cnv model
fragpattern model
fragsize model
Actual tissue
Predicted tissue

Supplementary Fig. 9. Performance of tissue-of-origin localization using samples predicte d as cancer. (A) Average accuracy (left) and accuracy (right) on each cancer type for differen t models on the MGI training cohort. **(B)** Average accuracy (left) and accuracy (right) on each cancer type for different models on the Illumina training cohort. **(C-D)** Confusion matrix for localization using the combined model on the **(C)** MGI training cohort and **(D)** Illumina traini ng cohort. The y-axis represents the actual site, and the x-axis represents the predicted site. The numbers in the cells of the matrix represent the proportion of samples of each cancer ty pe localized to respective tumor sites. **(A-D)** Our genome, epigenome, and combined models were compared with predictions based on fragmentation patterns[9] (fragpattern), fragment size profiles[8] (fragsize), and copy number variations[3] (cnv). Source data are provided as a Source D ata file.

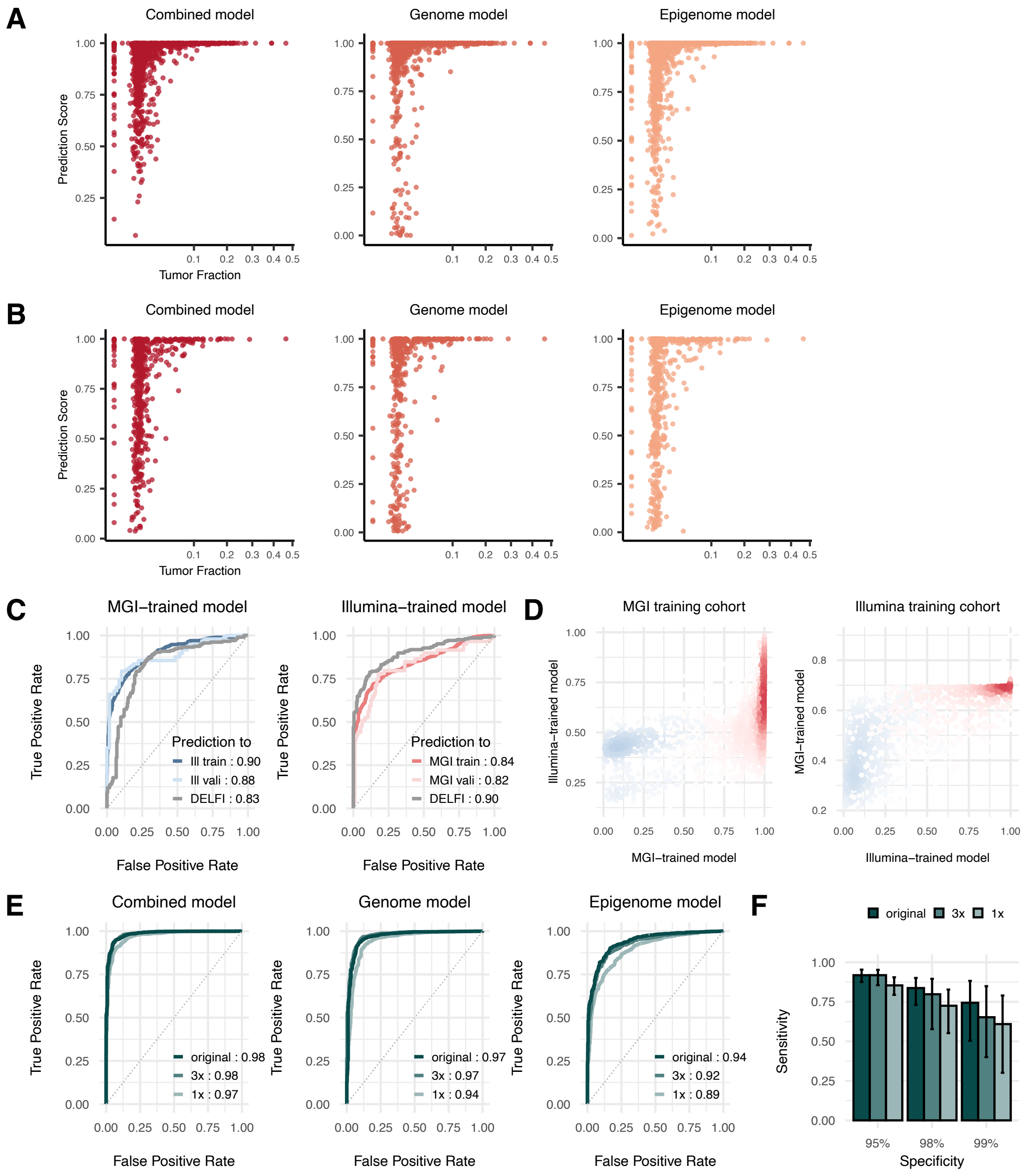
A
Combined model
Genome model
Epigenome model
Prediction Score
Tumor Fraction
B
Combined model
Genome model
Epigenome model
Prediction Score
Tumor Fraction
C
MGI−trained model
Illumina−trained model
True Positive Rate
False Positive Rate
Prediction to
Ill train : 0.90
Ill vali : 0.88
DELFI : 0.83
Prediction to
MGI train : 0.84
MGI vali : 0.82
DELFI : 0.90
D
MGI training cohort
Illumina training cohort
Illumina−trained model
MGI−trained model
E
Combined model
Genome model
Epigenome model
True Positive Rate
False Positive Rate
original : 0.98
3x : 0.98
1x : 0.97
original : 0.97
3x : 0.97
1x : 0.94
original : 0.94
3x : 0.92
1x : 0.89
F
original
3x
1x
Sensitivity
95%
98%
99%
Specificity

Supplementary Fig. 10. Additional analyses of our model. **(A-B)** Correlation between cfDNA tumor fraction and prediction score of combined, genome and epigenome model of the **(A)** MGI training cohort and **(B)** Illumina training cohort. **(C-D)** Combined models of the MGI training cohort and Illumina training cohort were used to predict the external cohort dataset. **(C)** ROC curve for the external cohort prediction. The combined model of the MGI training cohort was used to predict the Illumina training cohort, Illumina validation cohort and DELFI cohort (left). The combined model of the Illumina training cohort was used to predict the MGI training cohort, MGI validation cohort and DELFI cohort (right). **(D)** Density scatter plots which represent the relationship between the prediction score of the MGI trained model and the Illumina trained model on the MGI training cohort (left) and Illumina training cohort (right). The red dots indicate the density of cancer patient samples. The blue dots indicate the density of normal control samples. **(E-F)** Downsampled cfDNA WGS (3x, 1x) were compared with the original cfDNA WGS (5x) of the MGI training cohort. **(E)** ROC curve and **(F)** Sensitivity values with the 95% confidence Interval at 95%, 98%, 99% specificity for the different depth models of the MGI training cohort. Confidence interval for sensitivity value was calculated from 1,000 bootstraping samplings. Illu train, Illumina training cohort; Illu vali, Illumina validation cohort; MGI train, MGI training cohort; MGI vali, MGI validation cohort. Source data are provided as a Source Data file.

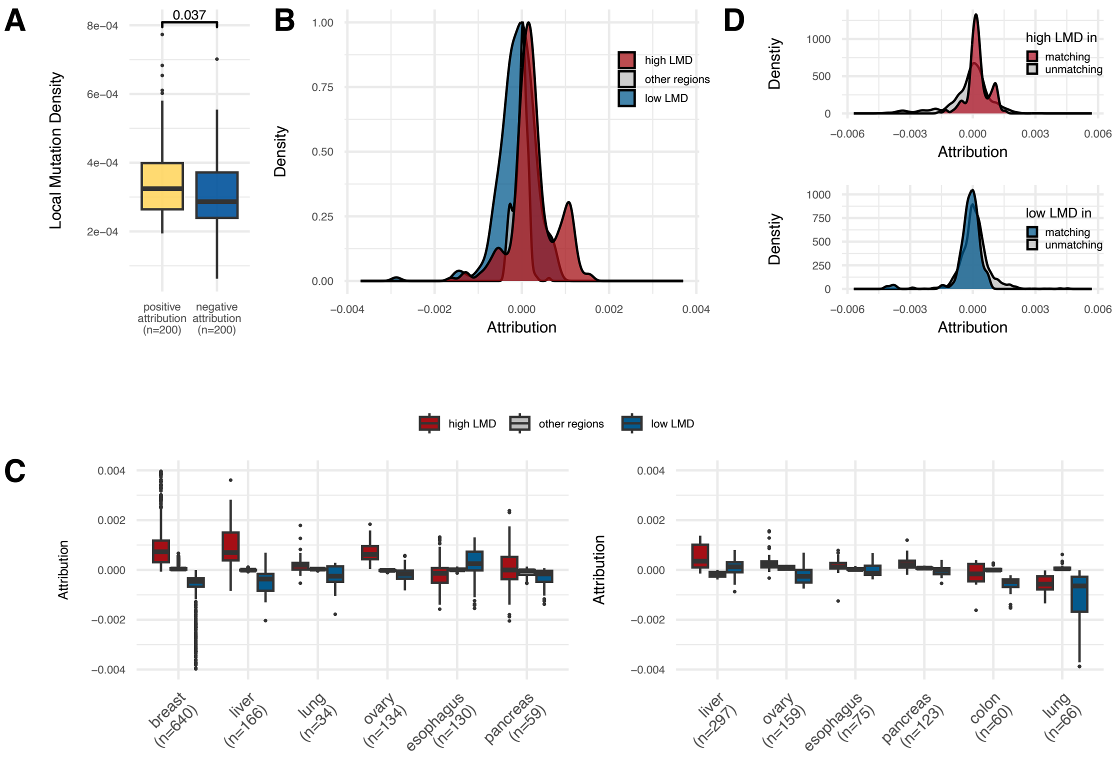
A
B
C
D
0.037
Local Mutation Density
8e−04
6e−04
4e−04
2e−04
positive attribution (n=200)
negative attribution (n=200)
Density
1.00
0.75
0.50
0.25
0.00
−0.004
−0.002
0.000
0.002
0.004
Attribution
high LMD
other regions
low LMD
Denstiy
high LMD in
matching
unmatching
low LMD in
1000
750
500
250
0
−0.006
−0.003
0.003
0.006
breast (n=640)
liver (n=166)
lung (n=34)
ovary (n=134)
esophagus (n=130)
pancreas (n=59)
liver (n=297)
ovary (n=159)
esophagus (n=75)
pancreas (n=123)
colon (n=60)
lung (n=66)

Supplementary Fig. 11. Additional interpretation of the genome model. (A) LMD values obtained from tumor tissues in the LVD regions with positive or negative attribution for cancer samples in the genome model for cancer detection on the Illumina training cohort. *P* values from the Wilcoxon test are indicated (two-sided). Each box indicates IQR and median, whiskers indicates 1.5 x IQR, black dots indicates outlier. **(B)** Distribution of the attribution values assigned by the cancer localization genome model of the Illumina training cohort to high or low LMD regions in comparison to other regions of the PCAWG cancer type matching the given prediction label. **(C)** The same plot as Fig. 5B and Supplementary Fig. 11B, broken down by cancer types for the MGI training cohort (left) and Illumina training cohort (right). Box elements are same as (A). **(D)** Comparison of the attribution values assigned by the cancer localization genome model to high (upper) or low (lower) LMD regions of the PCAWG cancer types matching versus unmatching with the given prediction label. Source data are provided as a Source Data file.

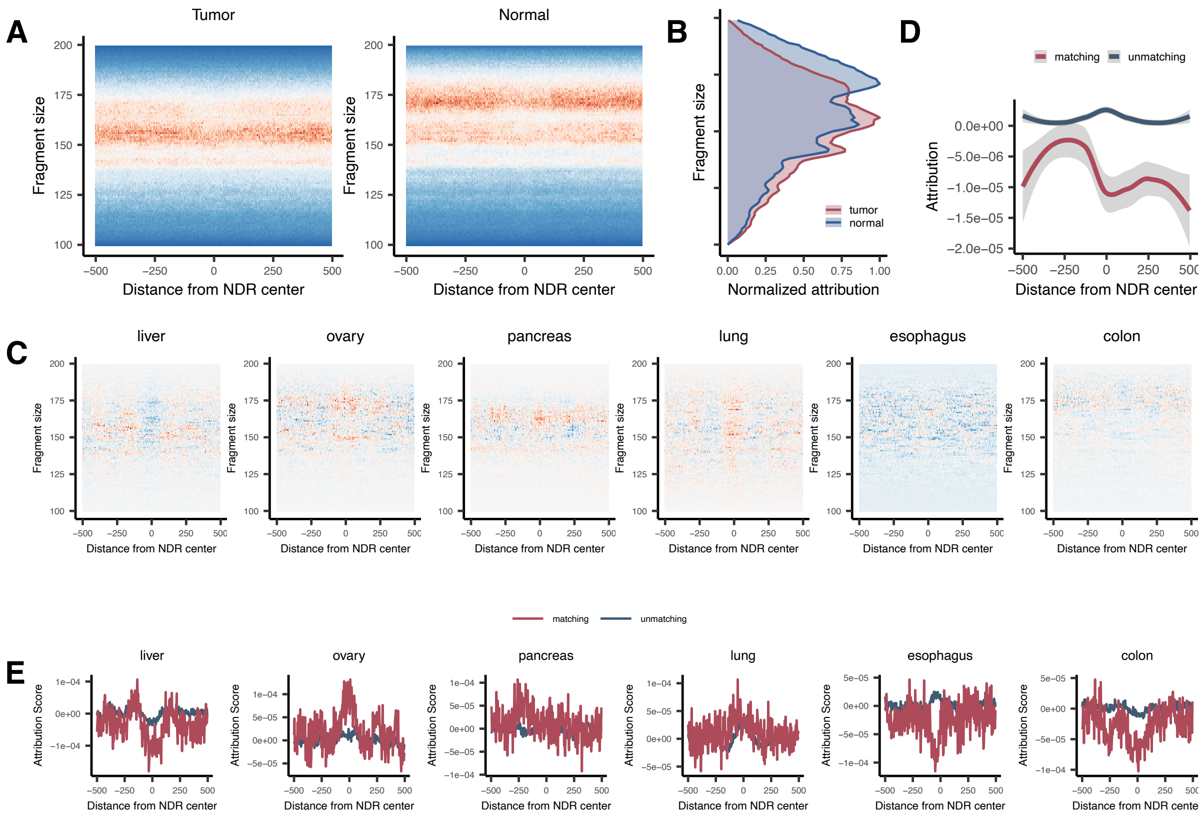
A
Tumor
Normal
Fragment size
Distance from NDR center
B
Fragment size
Normalized attribution
tumor
normal
D
matching
unmatching
Attribution
Distance from NDR center
C
liver
ovary
pancreas
lung
esophagus
colon
Fragment size
Distance from NDR center
E
matching
unmatching
liver
ovary
pancreas
lung
esophagus
colon
Attribution Score
Distance from NDR center

Supplementary Fig. 12. Additional interpretation of the epigenome model. (A) Attribution values of the tissue-specific V-plots of the epigenome model for cancer detection on the Illumina training cohort. The average attribution values across the tissue-specific V-plots are compared for cancer samples (left) and normal samples (right). **(B)** Distribution of the normalized attribution values according to fragment size. **(C)** Attribution values mapped to the tissue-specific V-plots of the epigenome model for tissue-of origin localization on the Illumina training cohort. The average attribution values across the tissue-specific V-plots are shown for each cancer type. **(D)** Attribution values of the epigenome model for cancer localization on the Illumina training cohort, as averaged across the NDRs of the matching (red) versus unmatching (blue) cancer types, and plotted according to the distance from the NDR midpoints. Attribution values were smoothed using lowess regression. **(E)** The sample plot as (D) broken down by cancer types. Source data are provided as a Source Data file.
