# Supplementary material for: Integrative modeling of tumor genomes and epigenomes for enhanced cancer diagnosis by cell-free DNA
Source: Nat Commun. 2023 Apr 10;14:2017. doi: 10.1038/s41467-023-37768-3 (PMC10085982; doi:10.1038/s41467-023-37768-3)
Supplement: Supplementary file 3 — Reporting Summary [file 41467_2023_37768_MOESM3_ESM.pdf]

nature portfolio

Corresponding author(s): Jung Kyoon Choi

Last updated by author(s): Jan 5, 2023

Reporting Summary

Nature Portfolio wishes to improve the reproducibility of the work that we publish. This form provides structure for consistency and transparency in reporting. For further information on Nature Portfolio policies, see our Editorial Policies and the Editorial Policy Checklist.

Statistics

For all statistical analyses, confirm that the following items are present in the figure legend, table legend, main text, or Methods section.

n/a	Confirmed	
☐	☒	The exact sample size (*n*) for each experimental group/condition, given as a discrete number and unit of measurement
☐	☒	A statement on whether measurements were taken from distinct samples or whether the same sample was measured repeatedly
☐	☒	The statistical test(s) used AND whether they are one- or two-sided<br>*Only common tests should be described solely by name; describe more complex techniques in the Methods section.*
☐	☒	A description of all covariates tested
☐	☒	A description of any assumptions or corrections, such as tests of normality and adjustment for multiple comparisons
☐	☒	A full description of the statistical parameters including central tendency (e.g. means) or other basic estimates (e.g. regression coefficient) AND variation (e.g. standard deviation) or associated estimates of uncertainty (e.g. confidence intervals)
☐	☒	For null hypothesis testing, the test statistic (e.g. *F*, *t*, *r*) with confidence intervals, effect sizes, degrees of freedom and *P* value noted<br>*Give P values as exact values whenever suitable.*
☒	☐	For Bayesian analysis, information on the choice of priors and Markov chain Monte Carlo settings
☒	☐	For hierarchical and complex designs, identification of the appropriate level for tests and full reporting of outcomes
☒	☐	Estimates of effect sizes (e.g. Cohen's *d*, Pearson's *r*), indicating how they were calculated

Our web collection on statistics for biologists contains articles on many of the points above.

Software and code

Policy information about availability of computer code

Data collection	All the softwares used in this study were described in Methods
Data analysis	Tensorflow(2.4.1), Scikit-learn(1.0.1), bwa-mem(0.7.17-r1188), GATK(4.2.0.0), Varscan2(v2.4.4), ichorCNA, HMMcopy, MACS2(2.2.7.1), HMMRATAC, Trim-galore(1.18), Cutadapt(1.18), samtools(1.7), Mutect2(4.2.0.0), edgeR, intersectBed(v2.29.2), XGBoost(1.5.1), LOWESS regression

For manuscripts utilizing custom algorithms or software that are central to the research but not yet described in published literature, software must be made available to editors and reviewers. We strongly encourage code deposition in a community repository (e.g. GitHub). See the Nature Portfolio guidelines for submitting code & software for further information.

Data

Policy information about availability of data

All manuscripts must include a data availability statement. This statement should provide the following information, where applicable:

- Accession codes, unique identifiers, or web links for publicly available datasets
- A description of any restrictions on data availability
- For clinical datasets or third party data, please ensure that the statement adheres to our policy

The 3,366 cfDNA WGS data generated in this study have been deposited in the European Genome-phenome Archive under accession code (EGAD00001009335). Our data are available under restricted access for protecting personal information. DELFI cfDNA WGS data also can be obtained from EGA (EGAD00001005339). Tumor somatic mutation MAF data were downloaded from PCAWG (https://dcc.icgc.org/releases/PCAWG/consensus_snv_indel/

final_consensus_passonly.snv_mnv_indel.tcga.controlled.maf.gz, https://dcc.icgc.org/releases/PCAWG/consensus_snv_indel/final_consensus_passonly.snv_mnv_indel.icgc.controlled.maf.gz). Tissue ATAC-seq bam files were downloaded from TCGA (https://portal.gdc.cancer.gov/). ATAC-seq of GM12878 and K562 were downloaded from SRA (GM128782 : https://www.ncbi.nlm.nih.gov/sra/?term=SRR891268, K562 : https://www.ncbi.nlm.nih.gov/sra/?term=SRR8137174). MNase-seq and histone modification ChIP-seq data were downloaded from ENCODE (GM12878 MNase-seq : https://www.encodeproject.org/files/ENCFF000VLH/@@download/ENCFF000VLH.bam, K562 MNase-seq : https://www.encodeproject.org/files/ENCFF000VMJ/@@download/ENCFF000VMJ.bam, GM12878 H3K27ac : https://www.encodeproject.org/files/ENCFF197QHX/@@download/ENCFF197QHX.bam, GM12878 H3K9ac : https://www.encodeproject.org/files/ENCFF415YCS/@@download/ENCFF415YCS.bam, GM12878 H3K4me1 : https://www.encodeproject.org/files/ENCFF753GZX/@@download/ENCFF753GZX.bam, GM12878 H3K4me2 : https://www.encodeproject.org/files/ENCFF794KPF/@@download/ENCFF794KPF.bam, GM12878 H3K4me3 : https://www.encodeproject.org/files/ENCFF375WTP/@@download/ENCFF375WTP.bam)

Human research participants

Policy information about studies involving human research participants and Sex and Gender in Research.

Reporting on sex and gender	There are no sex- and gender-based analysis in this study.
Population characteristics	Plasma samples form healthy individuals and patients with breast, hepatic, lung, ovarian, colorectal, pancreatic, bile duct, esophagus, or head and neck cancer were obtained from six institutions. All the healthy and patient samples were obtained under Institutional Review Board approved protocols with informed consent from all participants for research use at participating institutions.
Recruitment	All clinical data were retrospectively collected.
Ethics oversight	The study protocol was approved by the Institutional Review Board of each participating institution, and written informed consent was obtained from all patients before enrollment.

Note that full information on the approval of the study protocol must also be provided in the manuscript.

Field-specific reporting

Please select the one below that is the best fit for your research. If you are not sure, read the appropriate sections before making your selection.

[x] Life sciences [ ] Behavioural & social sciences [ ] Ecological, evolutionary & environmental sciences

For a reference copy of the document with all sections, see nature.com/documents/nr-reporting-summary-flat.pdf

Life sciences study design

All studies must disclose on these points even when the disclosure is negative.

Sample size	Sample size was not calculated in this study. Training was performed using MGI and Illumina cohorts, which both have sufficient number of samples (MGI: 1396 cancer vs 417 healthy, Illumina: 573 cancer vs 670 healthy).
Data exclusions	None.
Replication	On both MGI and Illumina cohorts, we used a cross-validation method during model training and also tested our model using independent validation data from a separate batch.
Randomization	We do not have experimental groups.
Blinding	Not relevant in this study. We designed retrospective study for cancer diagnosis.

Reporting for specific materials, systems and methods

We require information from authors about some types of materials, experimental systems and methods used in many studies. Here, indicate whether each material, system or method listed is relevant to your study. If you are not sure if a list item applies to your research, read the appropriate section before selecting a response.

Materials & experimental systems

n/a	Involved in the study
☒	☐ Antibodies
☒	☐ Eukaryotic cell lines
☒	☐ Palaeontology and archaeology
☒	☐ Animals and other organisms
☒	☐ Clinical data
☒	☐ Dual use research of concern

Methods

n/a	Involved in the study
☒	☐ ChIP-seq
☒	☐ Flow cytometry
☒	☐ MRI-based neuroimaging
