# Supplementary material for: Integrative modeling of tumor genomes and epigenomes for enhanced cancer diagnosis by cell-free DNA
Source: Nat Commun. 2023 Apr 10;14:2017. doi: 10.1038/s41467-023-37768-3 (PMC10085982; doi:10.1038/s41467-023-37768-3)
Supplement: Supplementary file 4 — Description of Additional Supplementary Files [file 41467_2023_37768_MOESM4_ESM.pdf]

Description of Additional Supplementary Files

File Name : Supplementary Data 1
Description : cfDNA data information

File Name : Supplementary Data 2
Description : Summary of patients and samples analyzed

File Name : Supplementary Data 3
Description : PCAWG WGS sample information

File Name : Supplementary Data 4
Description : High and low LMD regions per cancer type

File Name : Supplementary Data 5
Description : ATAC-seq sample information

File Name : Supplementary Data 6
Description : NDR peak count per sample

File Name : Supplementary Data 7
Description : Tissue-specific NDRs

File Name : Supplementary Data 8
Description : Results of cancer detection

File Name : Supplementary Data 9
Description : Results of tissue-of-origin localization

File Name : Supplementary Data 10
Description : Hyperparameter space
